# A Forest Bathing Intervention in Adults with Intellectual Disabilities: A Feasibility Study Protocol

**DOI:** 10.3390/ijerph192013589

**Published:** 2022-10-20

**Authors:** Elena Bermejo-Martins, María Pueyo-Garrigues, María Casas, Raúl Bermejo-Orduna, Ana Villarroya

**Affiliations:** 1 Department of Community, Maternity and Pediatric Nursing, School of Nursing, University of Navarra, Campus Universitario, 31008 Pamplona, Spain; 2 IdiSNA, Navarra Institute for Health Research, 31008 Pamplona, Spain; 3 Department of Environmental Biology, Faculty of Science, University of Navarra, Campus Universitario, 31080 Pamplona, Spain; 4 Grupo de Humanidades Ambientales, BIOMA, 31008 Pamplona, Spain

**Keywords:** forest bathing, intellectual disabilities, feasibility study, protocol, Shinrin-yoku

## Abstract

Forest bathing practices benefit individuals’ physical and mental health. A growing number of published studies provide evidence of such effects in diverse populations and contexts. However, no literature has been found that evaluates the effects of forest bathing on people with intellectual disabilities. In this paper, we present a quasi-experimental pre–post protocol for assessing the preliminary efficacy and feasibility of a forest bathing intervention in a group of adults with intellectual disability. An 11-weekly session program will be applied in the forests of the Ollo Valley, Navarre (Spain). The preliminary efficacy outcomes will be blood pressure, psycho-physiological coherence parameters and quality of life. The feasibility of the intervention will be assessed through data on barriers and facilitators of the implementation process and indicators of environmental comfort (physiological equivalent temperature and thermic perception). This study offers an opportunity for people with intellectual disabilities to benefit from a forest bathing intervention and explore its effects not only on their quality of life, but also on the improvement in their physiological and psychological state. This feasibility study is an essential step to explore crucial aspects for a future full-scale trial.

## 1. Introduction

Currently, 55% of the people worldwide live in urban rather than rural areas, with this number projected to reach 68% by 2050 [1]. This tendency increases the necessity for people to utilize forest and green spaces for health and well-being purposes [2], underlying the value of nature. The positive effects of forest ecosystems on human health have been proven by numerous investigations across diverse disciplinary fields [3,4,5,6]. Shinrin-yoku or forest bathing has become a global trend during the past decade as a reaction to the current flood of stimuli from and the hectic daily life in our modern society [2]. It refers to the practice of immersing oneself in natural forest environments by mindfully using one’s senses [7,8].

Accumulated evidence confirms that forest bathing has quantifiable positive outcomes for individuals’ physical and mental health [9,10,11,12,13]. The physiological health benefits include increases in the parasympathetic activity, which plays an important role in physical recovery [14]; the decrease of physiological biomarkers, such as cortisol levels and alpha-amylase; [15] and the lowering of heart rate and blood pressure [16,17]. In addition, forest bathing mitigates respiratory problems [6,18], sleep disorders [19], depression [20], and high levels of blood glucose [11]. Moreover, these practices have positive effects on the immune system and some inflammatory parameters [8,12] There is also a positive impact on psychological and mental health from reducing stress, symptoms of depression and anxiety, negative emotions, confusion, and fatigue [6,8,14,21]. In addition, forest bathing increases the feelings of awe, gratitude, and selflessness, while improving mental relaxation and increasing attentional focus [8,14,21].

The studies cited above cover a wide range of populations, including both healthy people and those with pre-existing conditions. Special attention has been paid to people suffering from chronic cardiovascular and respiratory diseases, diabetes, skin diseases, and a weakened immune system [2,11], and also to mental health problems such as depression or psychotic disorders [22,23]. However, we found no studies addressing adults with intellectual disabilities. Intellectual disability is a term used when there are limits to a person’s ability to learn at an expected level and function in daily life [24]. These individuals frequently experience health disparities, such as the failure to be included in public health efforts and other prevention activities. A sign of this might be the aforementioned gaps in the literature that reveal a lack of intervention trials [25], specifically in the realm of forest bathing. 

In the Spanish context, forest bathing investigations are recent and still scarce. Some examples are exploratory studies focused on patients with fibromyalgia [26], from the general healthy public [27], and after the COVID-19 lockdown [28,29]. Therefore, the main goal of this study is to provide information about the health effects of forest bathing in adults with intellectual disabilities. It also aims to provide insights for future research involving this target group.

## 2. Study Design

A quasi-experimental, pre–post design will be carried out following the methodological framework for complex interventions proposed by the Medical Research Council [30]. This framework is a very helpful guide to identifying the key questions about complex interventions, and to designing and conducting research with a diversity of perspectives and appropriate choice of methods. Complex intervention research can be considered in terms of phases, although they are not necessarily sequential: (1) development or identification of an intervention, (2) assessment of the feasibility of the intervention and evaluation design, (3) evaluation of the intervention, and (4) impact of its implementation. Therefore, this study corresponds with the assessment of feasibility phase of the intervention in order to make decisions about progression to the next stage of evaluation.

This study is registered at ClinicalTrials.gov (accessed on 26 July 2022) (identifier: NCT05472571).

### 2.1. Objectives

Specifically, this study aims to:

- Estimate the preliminary efficacy of an 11-session forest bathing intervention on adults with intellectual disability.
- Examine the feasibility (facilitators, barriers, unforeseen events, and indicators of environmental comfort) of applying the intervention to adults with intellectual disability.

### 2.2. Sample Size Determination

Conventional sample size calculation does not apply due to the exploratory nature of the investigation at this stage of the MRC framework, whose main objective is to explore the preliminary efficacy and feasibility aspects of the implementation process [31]. Nevertheless, we considered that a minimum of 30 would be sufficient to give a reasonable indication of the likely effect size in line with other similar papers [32,33,34]. Assuming attrition of 15%, the study seeks to recruit 35 persons (the number of eligible participants with intellectual disabilities enrolled in the occupational center where the study will be carried out).

### 2.3. Participants and Recruitment

**Figure 1:**
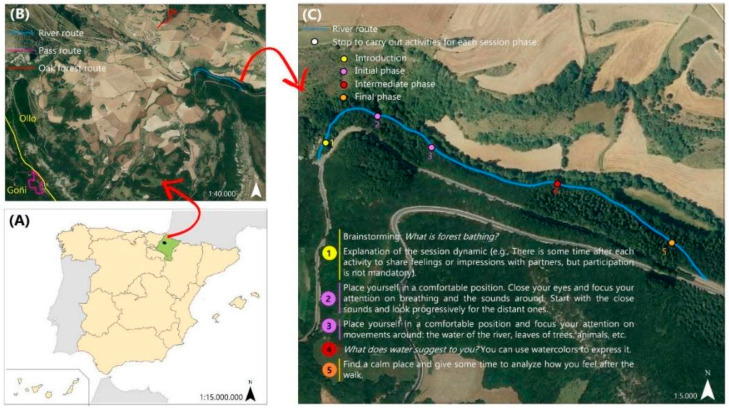
(**A**) Location of the Ollo valley (black dot) in Navarre (green). (**B**) Location of the three routes (river, in blue; oak forests, in red and pink). (**C**) Example of the distribution of phases and activities in a session following the river route (FTHub sequence selected for this intervention). Source: own elaboration from the cartography of IDENA (https://geoportal.navarra.es/es/mapas/cartografia, accessed on 10 August 2022).

The study will be conducted in a social cooperative that manages an occupational center and supervised apartments for 35 adults (aged 18–36) with low-to-moderate intellectual disability (and in some cases diagnosed with dual disability) in the Ollo valley (Navarra, Northern Spain) (see Figure 1A). Adults only enroll in this center after getting an official diagnosis by a team of doctors, psychologists and social workers from the regional Health System and Social Security System. The diagnostic criteria are those established in the national law [35]. Only adults from this occupational center will be eligible for this study. 

The eligibility criteria for participants are the following:

- Inclusion criteria: (1) adults with intellectual disability who are already enrolled in the activities of the participant occupational center; (2) able to read and/or speak in Spanish; and (3) with their own or legal guardian’s informed consent.
- Exclusion criteria: (1) adults with intellectual disability from other associated centers who are not enrolled in the daily activities of the participant occupational center; (2) adults with intellectual disabilities who may require intense and individual supervision from their educators throughout the activity.

Literacy or numeracy will not be required aspects for participants’ inclusion. To ensure their understanding of any information provided, reference educators will assist participants during the information process and data collection moments, to support those who face difficulties understanding written documents (information letter, consent form, or questionnaires). A person’s reference educator is that caregiver who usually works closely with him/her and acts as his/her mentor in the center. 

One month before starting the recruitment, there will be an informative meeting between the university’s project leaders and all workers (manager, educators, and technicians) and users from the social cooperative. Participants and their educators will have the opportunity to ask questions and concerns about the intervention to the researchers, the cooperative manager, and center’s workers. Once they express their willingness to participate, the center’s social worker and psychologist will conduct a preliminary screening to ensure the participants fulfill the inclusion criteria. Eligible participants will receive an invitation letter together with written information about the project and the informed consent document. Researchers’ contact information will be also provided to clarify any issues that might arise.

Besides the collaboration between the social cooperative and the university, an association of forest owners (Foresna-Zurgaia) and an environmental consultancy (Basartea) are also included in the partnership. They will be mainly responsible for mapping and conditioning the forest pathways to conduct the intervention.

## 3. Data Collection Plan

**Figure 2:**
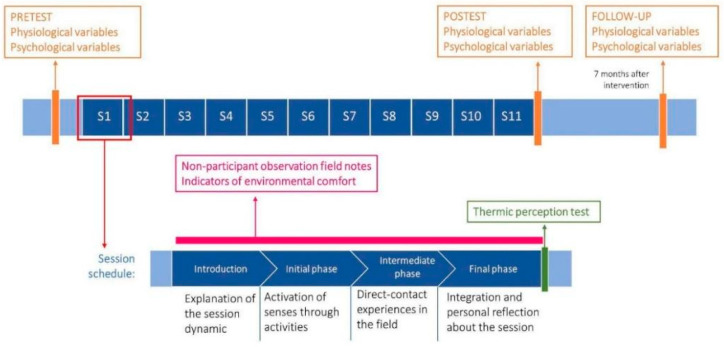
Intervention and data collection schedule. FTHub sequence selected for this intervention.

Once participants sign the consent, the baseline measurement will take place before the first forest bathing session (two weeks before the intervention). The same set of measures will be collected right after finishing the last session (posttest) and seven months after completing the intervention (follow-up) (Figure 2). The timelapse before the follow-up data collection responds to a study recommendation of gathering mid- and long-term measures after a forest bathing-based intervention [36,37]. Since this is an exploratory study, we will take just the mid-term follow-up measures, as we understand that the long-term ones correspond to a full study. Although the general duration recommendation for mid-term is 6 months after finishing the intervention [32], we will consider 1 month of grace period before or after the 6 months target assessment time to take into account significant events in context. 

Two researchers will collect the physiological and psychological data in one allocated room in the occupational center. This room will be a big open area with windows overlooking the forest and furnished with coaches, exercise mats, tables, chairs, office, and craft supplies. This room will be familiar to participants, as they will perform different daily activities and meetings there. The three data collection events will take place in the same room.

### 3.1. Outcomes, Measures and Instruments

First, sociodemographic variables will be collected, such as age, sex, disability grade, medication intake, mental comorbidities, geographical origin, and leisure time in contact with nature. Participants will fill out this data before starting the intervention. Then, preliminary efficacy outcomes will be estimated by psycho-physiological coherence (HRV parameters), blood pressure, and self-perceived quality of life measures.

#### 3.1.1. Blood Pressure

The instruments used to measure blood pressure will be three automatic upper-arm blood pressure monitors, using the same brand and functioning conditions. This device will always be placed on the left arm in a seated resting position and in order to detect any errors or abnormalities, the nurse researchers involved in this project will check out the results.

#### 3.1.2. Psychophysiological Coherence

The primary measure used to assess physiological or personal coherence is the heart rate variability (HRV) [32]. This parameter refers to the changes in the time interval between two consecutive heartbeats [38]. A coherent heart rhythm is defined as a relatively harmonic (sine wave-like) signal with a very narrow, high-amplitude peak in the low-frequency region (typically around 0.1 Hz) of the power spectrum with no major peaks in the other bands [39]. It is characterized by a heart rhythm pattern of elevated amplitude in low-frequency HRV. This coherence signal pattern is identified by a smooth, sinusoidal curve form in the heart rhythms [39]. While too much variability in heart rate can be harmful to efficient physiological functioning, too little variability correlates with a reduced capacity to adapt to environmental demands and stressors [38]. To estimate heart coherence, three parameters of the HRV are used widely:

- Time-domain [40]: to estimate the amount of variability in time intervals between successive interbeat (the standard deviation of all normal NN intervals (SDNN) and the root-mean-square analysis of successive interval differences (RMSSD);
- Frequency-domain: to estimate the distribution of the absolute or relative power (energy) into four frequency bands. High-frequency (HF) bands (0.15 to 0.4 Hz) are mainly affected by respiratory rhythms from 9 to 24 breaths per min (bpm) and reflect the parasympathetic activity. Low-frequency (LF) bands (0.04 to 0.15 Hz) are affected by baroreceptor activity and by respiratory rhythms from ~3 to 9 bpm; they reflect both sympathetic and parasympathetic activities. Very low-frequency (VLF) and ultra-low-frequency (ULF) (VLF, 0.0033 to 0.04 Hz; ULF, ≤0.0033 Hz) bands are spectral components with very low oscillations.
- Non-linear measures: to quantify the non-linear relationship between RR intervals (see [38] for more details).

The instrument to measure HRV parameters will be emWave^®^ Pro Plus device and software (5 min of registering). The emWave^®^ Pro Plus is a scientifically validated heart-rate monitoring system developed by the HeartMath Institute (Quantum Intech, Inc.) that facilitates HRV assessment. This device is very simple to use and completely harmless. It operates throughout a capillary sensor placed on the earlobe that registers heart pulse and rhythms information while users rest seated on a chair and breathe normally, in silence, for 5 min. When the assessment is completed, artifacts on the data can be checked and delated accordingly. It does not require calibration and operates both in Windows and Mac systems.

#### 3.1.3. Quality of Life

Regarding psychological variables, participants will fill in the Spanish version of the INICO-FEAPS Scale [41], aimed at assessing quality-of-life-related personal outcomes from the perspective of the person with an intellectual disability. It is a reliable tool for the target population, with an internal consistency of ∝ = 0.937 (Cronbach’s alpha) [41]. This self-report tool consists of 72 items assessed by a four-point Likert-type scale (from 1 = Never to 4 = Always) and distributed among 8 dimensions: self-determination (SD), rights (RI), emotional wellbeing (EMO), social inclusion (INCL), personal development (DEV), interpersonal relationships (RE), material wellbeing (MAT), and physical wellbeing (PHY). Each dimension is composed of nine items, and some scores are reversed because of the wording. Each subscale score is calculated by summing up the score of its nine items, and a total score is then calculated by summing up the subscale scores. Since subscales can be scored independently, we will only include in the study the four dimensions that we consider most appropriate to reflect the possible effects of a forest bathing intervention (EMO, DEV, RE, and PHY). This will be easier to handle for participants, especially those who have short attention spans. The reference educators will accompany participants when completing the questionnaire to provide support in case of doubts or difficulties in understanding. Cronbach’s alpha reliability for each sub-scale is the following: EMO (∝ = 0.792), DEV (∝ = 0.703), RE (∝ = 0.798), PHY (∝ = 0.659) [41].

### 3.2. Environmental Comfort

The physiological equivalent temperature (PET) value [42] and an individual questionnaire about thermic perception will be used to evaluate environmental comfort. Calculation of PET requires measuring ambient temperature and humidity, radiant temperature, wind speed, solar irradiance, and illuminance in the place where the activity happens [43], plus an estimation of user clothing and activity. An Ahlborn 2892-9 portable weather station fitted with a radiant temperature sensor (model FPA805GTS), a humidity and temperature sensor (model FHA646AG), and an actinometer (model FLA628S) will be used to record those environmental parameters during each session. The sensors will be set at 150 cm above ground level on an anchored tripod, and their readings integrated at 10-min intervals. The station will be concealed in close surroundings to the participants, to avoid distracting them from the activity. Registers within a radius of 50 m of the area of interest are considered valid. Regarding thermal sensation, participants will fill in a short questionnaire replicating tools used in published studies [44,45].

### 3.3. Fidelity, Barriers, and Facilitators of the Implementation Process

This data will be collected during the sessions in a non-participant observation field diary following an established common structure for each session report, including: (1) description of the session: name of the person in charge of the guidance, chosen route; (2) description of the ongoing process of the session: duration of each stage, an example of activities and materials delivered, and participants’ responses to them; (3) difficulties or climate considerations noticed during the activity or through participant comments. There will be two non-participant observers from the research team from the participant university and two from the center’s educational team.

## 4. Detailed Procedure

### 4.1. The Forest Bathing Intervention

The forest bathing intervention follows the method developed by Forest Therapy Hub [46] from the base approach proposed by Dr. Miyazaki and Dr. Li [47,48]. It consists of 11 consecutive sessions (one session per week), each one lasting about 2 h (Figure 2). Each session involves an easy walk through a forest area, interspersed with non-intrusive activities of contact with the surrounding nature aimed at fostering mindfulness and the use of the five senses. Three routes have been designed and prepared for their use with this population: two of them in oak forests and a third one next to a river (Figure 1B). To adapt the process to participants’ characteristics and needs, the sessions have been designed cooperatively by the center’s education team, technicians, psychologist and social worker, and the main researchers of this project. Therefore, the process of forest bathing is agreed upon by the whole team and customized in terms of time, routes, physical demand, and reflective questions during the activities. For example, the guides will help the participants through reflective activities to identify and share their thoughts, as this is usually a challenging process for many of them. Also, the team selected routes with very gentle or no slope and will prepare the path to minimize any possibility of stumbling or slipping while walking.

Sessions will be carried out in groups of 6 to 9 participants and guided by a trained guide (see training description and guides characteristics in Section 4.2). Although participants will experience different activities each week (Figure 1C), sessions will always follow the same sequence of four parts (Figure 2). The first phase introduces the activity, the setting, and the participants. Secondly, the “initial phase” focuses on disconnecting from every thought that is not the present moment. Thirdly, the “intermediate phase” is centered on connecting to the environment. Lastly, the “final phase” aims to integrate all the experiences of the previous phases. In all the phases, participants are invited to share their impressions with the rest of the group.

### 4.2. Forest Guide Training

A group of eighteen people has been trained as forest bathing guides. Ten of them are educators (responsible for programming the center’s pedagogical daily activities and supervising users), and eight are technicians (foresters and environmentalists) drawn from the other institutions involved in the project and familiar with the characteristics of the center and the people enrolled in it. The training consisted of a 96 h course led by an international expert in a professional capacity in forest bathing [46]. The content was focused on the design of forest bathing practices and on specific activities to carry out during the intervention, as well as some strategies to lead groups in this context. The training program involved five full-time days of theory and practice (40 h) in place and a practice period consisting in self-paced exercises, guiding two of the intervention's forest bathing sessions, and three follow-up online sessions to review the walks and to support the adaptations (56 h). We found no programs specifically tailored for guiding people with disabilities, so the guides were trained in forest bathing from a generic point of view in that sense. Since the cooperative’s educators already have the expertise in working with this type of individual, they will be the ones in charge of guiding the participant groups. The other guides (foresters and environmentalists) will act as support guides when necessary, always accompanied by an educator.

### 4.3. Ethics

The research ethics committee approval has been obtained from the University of Navarra Ethics Committee (code 2021.042). In the approval process, people with intellectual disabilities were considered to be a vulnerable population. For that reason, the information and consent documents were adapted to their needs by simplifying the writing where necessary and using accessible fonts and formats. Also, legal guardians were involved when applicable in the information and consent process. The permission to reach the center’s participants and workers for carrying out the study has also been obtained from the social cooperative through a signed formal letter. The emWave^®^ Pro Plus license has been purchased with the project funding and the INICO-FEAPS scale is an open-access instrument, and so the author’s permission has not been necessary. All participants will be given information about the study procedures and written consent will be obtained from subjects or their legal tutors before the project starts. Anonymity of participants will be safeguarded by assigning a participant code to each of them.

## 5. Expected Results

It is hypothesized that participants who complete the forest bathing intervention will report significantly:

- Higher quality of life.
- Reduced levels of blood pressure.
- Improved psycho-physiological coherence parameters.

## 6. Data Analysis Plan

R software will be used for quantitative data entry and analysis. The statistical analysis will follow a per protocol analysis approach, where missing data will not be included [49,50]. Descriptive statistics, including the frequency distribution and central tendency measures, will be used to summarize and describe the socio-demographic data. Sex, age, medication, and degree of intellectual disability will be analyzed as potential confounders. When normal distribution is achieved, a one-way ANOVA test with a level of significance at 5% will be performed to compare mean differences in paired data across the time. Otherwise, the Friedman test will be used as its non-parametric alternative. Effect size based on Cohen’s d [51] will be also calculated.

Qualitative data analysis for the observations registered in the field diary will be based on content analysis [52] where categories and thematic units will be identified.

## 7. Discussion

This protocol provides an example of a forest bathing intervention targeted at adults with intellectual disabilities, and the methodological route map for its application.

A range of aspects suggest this study’s novelty and relevance. First, forest ecosystems have been long suggested as potential providers of non-pharmacological health interventions. Research in this field has been translated directly into various initiatives worldwide [46,53,54,55] as forest bathing has become increasingly popular for promoting active lifestyles, being prescribed by healthcare professionals and recommended by national and local associations [9]. However, its beneficial effects have not yet been proved in adults with intellectual disabilities, which could entail some relevant considerations during the design and application of this type of intervention.

According to the suggestions provided by authors in the field of mindfulness, a common practice in forest bathing, there is a need to increase the work on the impacts and outcomes of integrated approaches to promoting mindfulness in intellectually disabled people [56]. The effectiveness of mindfulness has been evaluated in different populations for managing various physical and psychological health problems, including stress, anxiety, depression, pain, and eating disorders [57,58,59]. However, the use of mindfulness remains scarce in the broader demographic of individuals with intellectual disabilities [60], where the most common therapeutic approach is based on cognitive behavioral therapy [61].

Secondly, across most age groups, individuals with intellectual disabilities have higher rates of mental health conditions than the general population [62]. Indeed, up to a fifth of people with intellectual disabilities display challenging behavior that has a significant impact on their health and quality of life, requiring non-pharmacological treatments [63]. Meanwhile, forest bathing has demonstrated the important beneficial effects of reducing depression symptoms [36,37], decreasing anxiety, and increasing HRV parameters [64], but none of them have been assessed in this group. Similarly, measuring psycho-physiological coherence (including HRV) seems to be a major step forward in clinical research for intellectually disabled individuals, since variations on its parameters precedes observed behavioral excitation or agitation. Thus, these parameters may allow recognizing or predicting such patterns [65] and estimating the intervention effects more objectively. In the same line, positive associations have been found between quality of life, emotion regulation strategies, and HRV in people with intellectual disabilities [66], being considered a key psycho-physiological marker of mental and physical wellbeing.

Lastly, environmentalists, public health professionals, and tourism planners alike have recognized that there is an increased need to promote more sustainable approaches to enhancing human health and wellbeing through encouraging the use of natural environments [6,67]. This is especially the case due to more frequent and longer stays in forests having a stronger and more lasting effect than isolated and shorter visits [12].

Therefore, this intervention responds to a health equity framework and offers an important social perspective in addressing wellbeing and quality of life through nature in adults with intellectual disabilities. Using forest recreation as a community asset may help reduce stereotypes and empower this group to realize their full potential. Additionally, measuring psychological outcomes (such as quality of life) jointly with physiological parameters (such as HRV), external mediators’ factors (the environmental comfort), and qualitative data about the barriers and facilitators during the application process are other additional strengths of the study that will help future research lines.

### Limitations

Despite the abovementioned strengths, some important limitations of this study must be considered. Since the study tackles a very specific population, it may be hard to reach a sample size large enough to detect statistically significant differences across time in the intervention group. Also, the quasi-experimental, pre–post design entails the lack of a control group to compare with, which is a clear weakness in determining causality. However, due to the exploratory condition of this study, both aspects are not as crucial as they are in subsequent confirmatory trials [68].

## 8. Conclusions

This study could support the relevance of addressing the benefits of forest bathing on the physical and psychological health of adults with intellectual disabilities. Nevertheless, developing and evaluating this type of intervention entails several methodological challenges that need to be tested first in a feasibility study before later scaling up to a final trial. This study offers a promising and innovative strategy to promote health in adults with intellectual disabilities from a universal and positive approach.

## Data Availability

Not applicable.
